# Clearance of amyloid plaque via focused ultrasonication in a mouse model of Alzheimer's disease

**DOI:** 10.7150/thno.122123

**Published:** 2026-01-01

**Authors:** Songmin Lee, Jeungeun Kum, Kyeonghwan Kim, Tae Young Park, HeeEun Ko, Duk L. Na, Suk Yun Kang, Hyungmin Kim, YoungSoo Kim, Jaeho Kim

**Affiliations:** 1 Department of Pharmacy and Yonsei Institute of Pharmaceutical Science, College of Pharmacy, Yonsei University, Incheon, Republic of Korea.; 2 Bionics Research Center, Biomedical Research Division, Korea Institute of Science and Technology, Seoul, Republic of Korea.; 3 Happy mind Clinic, Seoul, Republic of Korea.; 4 Department of Neurology, Samsung Medical Center, Sungkyunkwan University School of Medicine, Seoul, Republic of Korea.; 5 Department of Neurology, Dongtan Sacred Heart Hospital, Hallym University College of Medicine, Hwaseong-si, Gyeonggi-do, Republic of Korea.

**Keywords:** amyloid-β, focused ultrasound, non-invasive brain stimulation, 5XFAD transgenic mouse

## Abstract

**Background:** The success of anti-amyloid-β (Aβ) monoclonal antibodies in recent clinical trials validates the promising approach of clearing amyloid-β in Alzheimer's therapy. Building on these successes, focused ultrasound (FUS), a non-invasive therapeutic modality that delivers acoustic energy to targeted brain regions with high precision, has emerged as a potential technique to modulate Aβ pathology, either in combination with drugs or as a standalone treatment. This study focused on the standalone potential of FUS to reduce Aβ plaques without accompanying drugs.

**Methods:** Synthetic Aβ42 aggregates were prepared and exposed to FUS. The changes in fibril and oligomer levels were analyzed using Thioflavin T (ThT) fluorescence, gel electrophoresis combined with photo-induced cross-linking of unmodified protein (PICUP) chemistry, transmission electron microscopy (TEM), and immunoblotting. The effect of FUS on Aβ42-induced cytotoxicity was evaluated in SH-SY5Y human neuroblastoma cells. FUS-mediated dissociation of Aβ plaques was performed by *ex vivo* and *in vivo* methods on the 5XFAD mouse model. In the *ex vivo* experiment, FUS was applied to brain slices, specifically targeting the hippocampal region. In the *in vivo* experiment, the left hippocampus of awake animals was sonicated in a transcranial manner ten times over two weeks using a miniature ultrasound transducer affixed to the skull. For both *ex vivo* and *in vivo* experiments, immunohistochemistry was performed on brain sections for measuring Aβ plaques after sonication. Blood was collected from animals before and after *in vivo* stimulation for plasma analysis.

**Results:** *In vitro*, FUS treatment reduced the β-sheet structure of synthetic Aβ42 aggregates by up to 55.28% in the ThT assay, and fibrillar Aβ42 levels by up to 62.27% in the gel electrophoresis, as further confirmed by TEM imaging, which showed disrupted fibrillar structures. The level of oligomeric Aβ42 was also reduced by up to 65.02% following FUS exposure. SH-SY5Y cells treated with FUS-treated Aβ42 aggregates exhibited improved viability from 81.56% to 90.48%, showing a tendency of attenuated Aβ42-induced cytotoxicity by FUS. *Ex vivo* FUS stimulation significantly reduced the number of Aβ plaques in the hippocampal region compared to untreated brain slices. *In vivo* transcranial FUS reduced both the number and size of plaques in the FUS-treated hippocampal and thalamic region compared to the contralateral side. Plasma analysis with Aβ42 enzyme-linked immunosorbent assay revealed a 65.91% increase in Aβ levels following FUS treatment compared to pre-treatment levels, suggesting that Aβ plaques dissociated by FUS were released into the bloodstream.

**Conclusions:** FUS exposure effectively reduced amyloid plaques in both *ex vivo* and *in vivo* models by disrupting fibrillar and oligomeric Aβ, demonstrating its potential as a non-invasive strategy for Aβ clearance.

## Background

Alzheimer's disease (AD) is a progressive neurodegenerative disorder characterized by cognitive decline, memory impairment, and functional deterioration of the brain 1, 2. A defining pathological hallmark of AD is the accumulation of amyloid-β (Aβ) peptides in the brain, which aggregate to form insoluble plaques that disrupt neuronal networks and ultimately lead to cell death 3. Thus, Aβ clearance has emerged as a pivotal therapeutic strategy in AD for modifying disease progression 4. Recently, monoclonal antibody therapies such as lecanemab and donanemab received approval from the U.S. Food and Drug Administration (FDA) for their ability to reduce amyloid burden in the brain 5-7. Despite their promise, these therapies are associated with serious adverse effects, including cerebral edema and microhemorrhages, underscoring the urgent need for safer, effective alternatives to mitigate Aβ pathology in AD 8, 9.

Focused ultrasound (FUS) is a non-invasive therapeutic technique that employs precisely targeted acoustic energy to treat specific brain regions 10, 11. Recently developed AD treatment techniques aim to modulate cholinergic pathways, drug delivery via cerebral spinal fluid (CSF), and temporal change of blood-brain barrier (BBB) permeability 12, 13. Initial studies in AD primarily investigated the use of FUS, typically in combination with intravenously administered microbubbles, to temporarily open the BBB, thereby facilitating enhanced delivery of anti-amyloid drugs to the brain 14, 15. Subsequent research revealed that FUS-induced BBB opening alone, without using additional anti-amyloid drugs, can also reduce Aβ fibrils and recover cognitive function in both animal models and AD patients 16, 17. BBB opening has been shown to improve lymphatic clearance of Aβ in 5XFAD mouse model 18. Furthermore, repeated bilateral sonication using FUS reduced both Aβ plaques and tau protein in the 3xTg-AD mouse model, and decreased amyloid levels in human AD patients 19. More recent studies have taken this a step further, demonstrating that FUS alone, without microbubble or BBB opening, can improve drug delivery by disrupting non-covalent interactions between plasma proteins and therapeutic agents, thereby increasing their bioavailability 20. Notably, these studies also showed that FUS can directly disrupt Aβ fibrils, suggesting its potential as a promising standalone strategy for targeting and dissociating amyloid plaques.

Building upon aforementioned emerging evidences, our study explores the direct application of FUS without BBB opening or pharmacological co-intervention, focusing solely on its capability to dissociate Aβ aggregates. We investigated FUS-induced Aβ dissociation through *in vitro*, *ex vivo,* and *in vivo* experiments using synthetic Aβ42 peptides and 5XFAD transgenic mice. In the *in vitro* experiments, synthetic Aβ42 aggregates were exposed to FUS to confirm the physical disruption of Aβ aggregates by FUS. In the *ex vivo* experiments, brain tissue containing plaques was collected and treated with FUS to evaluate its potential for clearing amyloid plaques. In the *in vivo* experiments, FUS was applied to the hippocampal region over two weeks to assess its feasibility as a therapeutic intervention. Both *ex vivo* and *in vivo* approaches resulted in a significant reduction in plaque burden, and an increase in plasma Aβ42 levels was observed *in vivo*, indicating plaque clearance. These findings highlight the therapeutic promise of FUS in targeting amyloid pathology.

## Methods

### FUS stimulation setup

The sonication setup consisted of a function generator (33510B, Keysight Technologies, USA) and a linear radio frequency (RF) power amplifier (240L, Electronics and Innovations, USA). In brain slice experiments, a single-element focused ultrasound with a 500 kHz center frequency (GPS500-D19-P38, The Ultran Group, USA) was used. For *in vivo* transcranial focused ultrasound (tFUS) stimulation in awake animals, an in-house built single-element miniature transducer with a 450 kHz center frequency was used. The intensity maps of both transducers were measured in degassed, deionized water using a needle-type hydrophone (HNR-0500, ONDA Corp., USA). The full-width at half-maximum (FWHM) dimensions of the acoustic intensity field for the 500 kHz transducer were approximately 7 mm in the lateral direction and 50 mm in the axial direction. The FWHM of the acoustic intensity field of the 450 kHz transducer was approximately 1.5 mm in the lateral direction and 4 mm in the axial direction.

In the brain slice stimulation, the protocol was as follows: 100 ms pulse duration (PD), 10% duty cycle (DC), 1 Hz pulse-repetition frequency (PRF), 1 s pulse repetition interval (PRI), 5 W/cm^2^ spatial-peak pulse-average intensity (I_SPPA_), and 0.5 W/cm^2^ spatial-peak temporal-average intensity (I_SPTA_) for 30-minute pulse train duration (PTD). In the *in vivo* awake tFUS stimulation, the protocol was as follows: 100 ms PD, 10% DC, 1 Hz PRF, 1 s PRI, 1.75 W/cm^2^ I_SPPA_ and 0.175 W/cm^2^ I_SPTA_ for 30-minute PTD. The parameters are reported following the ITRUSST consensus for standardized reporting of transcranial ultrasound stimulation 21. I_SPPA_ and I_SPTA_ were calculated as follows 22:



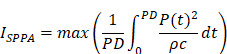





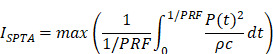



* *P*, pressure; *ρ*, density of the propagating medium; *c*, speed of sound in the propagating medium; *PRF*, pulse repetition frequency; *PD*, pulse duration.

### Acoustic simulation

Acoustic simulations were conducted using the k-Wave MATLAB toolbox to estimate the distribution of acoustic intensity within the brain 23. A 450 kHz transducer for awake tFUS stimulation with specifications identical to those outlined in the FUS stimulation setup was employed, featuring a width of 13 mm and a radius of curvature of 5.5 mm. The simulation domain was defined as 150 × 150 × 130, with a grid resolution of 20 points per wavelength (equivalent to 0.16 mm spacing). Ultrasound waves were applied for 100 μs to allow sufficient time for the focal point to fully form, and the Courant-Friedrichs-Lewy number was set to 0.05.

To model the geometry of the mouse skull, a statistical parametric mapping-based template was utilized 24. Regions with voxel intensities exceeding 1500 Hounsfield units were segmented as skull, excluding the diploe structure from the model. Both the skull and transducer were assumed to be submerged in water for the simulation 25. The acoustic properties defined for the simulation included water (speed of sound: 1482 m/s, density: 1000 kg/m³) and skull tissue (speed of sound: 2442 m/s, density: 1969 kg/m³) 26. Skull attenuation coefficients were not factored into the calculations.

Figure 4E and 4F show 2D cross-sectional views of the regions within the brain exhibiting the highest acoustic intensity. The simulation determined that the I_SPPA_ reached 1.22 W/cm², representing a reduction of approximately 30% compared to the free water intensity of 1.75 W/cm².

### *In vitro* and *ex vivo* FUS setup

A 35 mm imaging dish with a polymer coverslip bottom (μ-dish, 35 mm, low, ibidi GmbH, Germany) was used for FUS stimulation. A customized ultrasound guide was attached to the front of the transducer to mount the imaging dish. The guide was designed to secure the dish precisely at the transducer's focal point, ensuring accurate ultrasound stimulation delivered to the sample placed on the dish. The guide, combined with the transducer, supports the dish from below, allowing the ultrasound beam to be delivered vertically upward through the tissue.

### *In vitro* FUS experiment

Aβ42 peptides were synthesized using the dimethyl sulfoxide (DMSO)-incorporated fluorenylmethyloxycarbonyl chloride (Fmoc) solid-phase peptide synthesis (SPPS) as previously reported 27. Aβ42 peptides were dissolved in DMSO to prepare a 1 mM stock solution, which was stored at -80 °C until use. The Aβ42 stock was thawed and immediately diluted in a sodium phosphate buffer (10 mM sodium phosphate buffer with 100 mM NaCl, pH 7.4) to prepare a 25 μM Aβ42 solution. Aβ42 solutions were incubated for 2 days with agitation (400 rpm) at 37 ℃ to induce aggregation. Aβ42 aggregates were placed on an ibidi dish (80136) and exposed to FUS for 30 min. Non-incubated Aβ42 solution and non-FUS-treated Aβ42 aggregates were used as controls.

### Thioflavin T (ThT) assay

A 5 μM ThT (T3516, Sigma, USA) solution was prepared in 50 mM glycine buffer (pH 8.5) and protected from light until use. Aliquots of 25 μL of each sample were loaded into a 96-well half-area black flat-bottom microplate (3694, Corning, USA) and mixed with 75 μL of ThT solution. After incubation for 5 min at room temperature, fluorescence intensity was measured at an excitation/emission wavelength of 450/480 nm using an Infinite^®^ 200 PRO microplate reader (TECAN, Switzerland). All experiments were performed in triplicate.

### Immunoblot assay

For western blot with photo-induced cross-linking of unmodified protein (PICUP) chemistry, 1 mM Tris(2,2-bipyridyl)dichlororuthenium(II) hexahydrate (RuBpy; 544981, Sigma, USA) and 20 mM ammonium persulfate (APS; 431532, Sigma, USA) were prepared in 0.1 M sodium phosphate buffer (pH 7.5). Each 10 μL of the Aβ sample was mixed with 1 μL of each RuBpy and APS solution. The mixture was irradiated with visible light three times for 1 s, with 1-s intervals between irradiations. After irradiation, 3 μL of 5x sample buffer containing 10% (w/v) dithiothreitol (DTT) was added. Each 15 μL of sample was loaded onto a 4-20% gradient polyacrylamide gel (4561095, BIO-RAD, USA) and electrophoresed at 150 V. Proteins were transferred onto a PVDF membrane (1620177, BIO-RAD, USA) at 100 V for 30 min, and the membrane was blocked with 5% (w/v) skim milk in tris-buffered saline containing 0.1% Tween 20 (TBST) for 1 h at room temperature. The membrane was probed with 6E10 anti-Aβ antibody (SIG-39320, BioLegend, USA; 1:10,000), followed by a horseradish peroxidase (HRP)-conjugated goat anti-mouse IgG (H+L) secondary antibody (115-035-003, Jackson ImmunoResearch, UK; 1:50,000). For the oligomer dot blot assay, Aβ42 samples were spotted twice (2 μL each) onto a nitrocellulose membrane, with the second spot applied after the first had completely dried. The membrane was dried for 30 min, blocked with 5% (w/v) skim milk in TBST for 1 h at room temperature, and probed with anti-amyloid oligomer A11 antibody (AHB0052, Invitrogen, USA; 1:1500) followed by HRP-conjugated goat anti-rabbit IgG (H+L) (111-035-003, Jackson ImmunoResearch, UK; 1:15,000). Signals were developed using SuperSignal™ West Pico PLUS Chemiluminescent Substrate (34580, ThermoFisher, USA) and visualized with the FUSION Solo S software program.

### Transmission Electron Microscope (TEM)

Carbon-coated copper grids (CF200-CU, YMS, Korea) were glow-discharged using a PELCO easiGlow (Ted Pella, USA). Aβ42 samples were applied to each grid and incubated for 1 min. Excess solution was removed with filter paper, and the grids were negatively stained with 5 μL of 2% (w/v) uranyl acetate for 10 s. After complete drying, the samples were imaged using a Talos L120C (ThermoFisher, USA)*.*

### Cell viability assay

For the viability assay, Aβ42 solutions were prepared by diluting 5 mM Aβ42 DMSO stock to 25 μM with sodium phosphate buffer. The Aβ42 samples were incubated for 2 days at 37 ℃ with agitation (400 rpm) and subsequently exposed to FUS for 30 min. The aggregates were then diluted in high-glucose Dulbecco's Modified Eagle Medium (DMEM) media (LM001-05, WELGENE, Korea) to prepare 10 μM Aβ solutions. SH-SY5Y human neuroblastoma cells were cultured in growth media consisting of high-glucose DMEM supplemented with 10% FBS (A5256701, Gibco, USA) and 1% Penicillin-Streptomycin (15140122, Gibco, USA) in a humidified incubator with 5% CO_2_ at 37 °C. Cells were seeded at a density of 1.5 x 10^4^ cells/well in a 96-well cell culture plate (CLS3596, Corning, USA) and incubated for 12 h. After a 4-h serum starvation period, cells were treated with Aβ42 solutions for 12 h. Cell viability was assessed using D-Plus™ CCK cell viability assay kit (Eubiogene, Korea) according to the manufacturer's instructions.

### Animals

Male B6SJL-Tg(APPSwFlLon,PSEN1*M146L*L286V)6799Vas/Mmjax (5XFAD, MMRRC Strain #034840-JAX) mouse (n = 6) and a female B6SJLF1/J (Strain #100012) mouse (n = 1) were obtained from the Jackson Laboratory (USA) to establish a breeding colony. Offspring were weaned at 3 weeks of age and separated into transgenic and wild-type groups based on genotyping results. All mice were housed in groups of 4-5 per cage at the Yonsei University animal facility (Seoul, Korea) under controlled temperature and humidity conditions, with a 12:12-h light-dark cycle and *ad libitum* access to food and water. Age-matched littermates from the same generation were used for the same experiments. The number of mice in each group varied depending on the availability of the experimental animals.

### *Ex vivo* FUS experiment

The 14-month-old female 5XFAD mouse was sacrificed and perfused with 0.9% saline. Brain tissue was then collected and fixed overnight in ice-cold 4% paraformaldehyde at 4 °C. Following fixation, the tissue was immersed in 30% sucrose for 24 h and subsequently cut into 25 µm sections using a cryostat at -20 °C (Leica CM1860, Leica, Germany). Each brain slice was mounted at the center of the dish, and the guide cone and dish were filled with degassed water for acoustic coupling. An ultrasound absorber (Aptflex F48, Precision Acoustics, UK) was mounted on the top of the dish to minimize acoustic reflection. Sequentially collected brain slices were symmetrically mounted to facilitate direct comparison of the same plaque. One side of each paired brain slice was exposed to FUS for 30 min. Following exposure, the brain slices were processed for immunostaining for further analysis.

### Surgical procedures and experimental setup of the *in vivo* awake tFUS stimulation

5XFAD mice (11-month-old female, n = 5) were used for this study. Animals were anesthetized via an intraperitoneal injection of a ketamine/xylazine mixture (80 mg/kg ketamine, 10 mg/kg xylazine). An additional dose of anesthetic agent (one-third of the original dose) was administered as needed during surgical procedures. The head of the mouse was held using an adaptor (68014, RWD, China). After the scalp fur was removed, a midline incision was made to expose the skull. The skull surface was cleaned with saline solution and dried for transducer fixation.

A single-element miniature transducer with a 5 mm diameter was placed and secured with cyanoacrylate glue on the skull surface above the left motor cortex (Anterior-posterior (AP): 0 mm; Medial-lateral (ML): 1.5 mm) for transcranial sonication of the focused ultrasound. Dental acrylic cement (Vertex self-curing, Vertex-dental, NL) was additionally used for the fixation of the transducer. After the acrylic cement was cured, the scalp incision was sutured to cover the skull surface, leaving the connector part of the transducer exposed. Animals were allowed to recover for one week before the first experiment.

After recovery, the transducer was connected to the sonication system during stimulation. Mice underwent stimulation five times per week for two weeks, with each session lasting 30 min. Prior to the initial exposure, blood samples were collected via the lateral saphenous vein. After two weeks of stimulation, blood was collected within 10 min following the final session, and brain tissue was harvested.

### Histochemistry of brain sections

The brain was coronally sliced into 25 μm sections, and two slides per mouse were used for plaque analysis. The brain sections on glass slides were washed with phosphate-buffered saline (PBS) solution. For antigen retrieval, the slides were soaked in 1% sodium dodecyl sulfate (SDS) in PBS for 10 min and then blocked with 5% goat serum in PBS for 1 h at room temperature to prevent non-specific binding. Primary antibodies diluted in PBS with 5% goat serum were treated for 2 h at room temperature. Fluorescent secondary antibodies diluted in PBS were treated for 1 h at room temperature. Antibodies used in this study were 6E10 (SIG-39320, Biolegend, USA; 1:200), anti-glial fibrillary acidic protein (GFAP) antibody (AB5541, Sigma, USA; 1:300), Alexa Fluor™ 555-conjugated goat anti-mouse IgG (H+L) antibody (A21424, Invitrogen, USA; 1:200), and Alexa Fluor™ 568-conjugated goat anti-chicken IgY (H+L) antibody (A11041, Invitrogen, USA; 1:300). All steps following the secondary antibody treatment were performed in the dark. Thioflavin S (ThS; T1892, Sigma, USA) staining was performed after the 6E10 staining. ThS was diluted in 50% ethanol to make a final concentration of 0.015% and briefly sonicated. Slides were incubated with the ThS solution for 7 min at room temperature, followed by sequential wash with 80% ethanol twice and Milli-Q water twice for 30 s each. After nuclear staining using Hoechst 33342 (10 mg/L; ThermoFisher, USA) for 3 min at room temperature, slides were coverslipped using mounting media (Biomeda, USA) and dried overnight. Images were obtained using a VS200 fluorescence slide scanner (Evident, Japan) and a DM2500 fluorescence microscope (Leica, Germany). Aβ plaques were analyzed using Fiji software 28. Additionally, Hematoxylin and Eosin (H&E) staining was performed according to the manufacturer's instructions using a H&E staining kit (Abcam, UK).

### Enzyme-linked immunosorbent assay (ELISA)

To detect and quantify the levels of human Aβ42 in plasma, an ELISA was conducted using a human Aβ42 ultrasensitive ELISA kit (Invitrogen, USA) following the manufacturer's protocol. Briefly, plasma and CSF samples were prepared by diluting them up to 1:5 fold, respectively, in standard diluent buffer containing a protease inhibitor cocktail. Serial dilutions of the human Aβ42 standard were prepared at the following concentrations: 100, 50, 25, 12.5, 6.25, 3.13, 1.56, and 0 pg/mL. The standards and diluted samples were added to the appropriate wells of the plate. Next, the human Aβ42 detection antibody was added to the wells and incubated overnight at 4 °C. After washing the plate with wash buffer, a secondary antibody (anti-Rabbit IgG horseradish peroxidase) was added and incubated for 45 min at room temperature. The wells were then washed, and a chromogen solution was applied to the plate for 30 min in the dark. Finally, a stop solution was added, and the absorbance was measured at 450 nm using a SpectraMax M2 microplate reader (Molecular Devices, USA).

### Statistical analysis

One-way ANOVA followed by Bonferroni's multiple comparison tests was used for comparisons among multiple groups. An unpaired t-test was applied for comparisons between two groups, and a paired t-test for comparisons between paired samples *in vivo* (*p < 0.05, **p < 0.01, ***p < 0.001, ****p < 0.0001; other comparisons were not significant). Statistical analyses were performed using GraphPad Prism 10 software. Data are presented as mean ± standard error of the mean (SEM).

## Results

### FUS-induced disaggregation of Aβ42 fibrils *in vitro*

To examine whether FUS disrupts the Aβ assemblies *in vitro*, we utilized the Aβ42 peptide, which is the most aggregation-prone and pathogenic Aβ isoform in AD 29. Synthetic Aβ42 peptides were incubated for 2 days at 37 ℃ with agitation to induce self-aggregation, followed by exposure to FUS with three different parameters (P1-P3) (Figure 2A). We first quantified the amount of β-sheet structures by ThT, which emits fluorescence upon binding to them (Figure 2B). During 2-day incubation, β-sheet structures had 13-fold increased (non-incubated Aβ42, 0d; 7.72%). Across P1-P3, ThT fluorescence intensities decreased by 46.14, 36.62, and 55.28%, respectively, compared to the non-sonicated 2-day aggregated control (2d; 100%). Notably, the fibrillar reduction was not relative to the applied FUS intensity, suggesting that the disruption depends on the complicated coordination of acoustic conditions.

To assess FUS-induced alterations in the size distribution of Aβ42 aggregates, we performed sodium dodecyl sulfate polyacrylamide gel electrophoresis (SDS-PAGE) combined with PICUP chemistry, which preserves non-covalent assemblies via radical reaction (Figure 2C). We found that protofibril to fibrillar Aβ42 around ~250 kDa (≈55-mer; fAβ) was prominently reduced following FUS, with decreases of 40.24, 62.27, and 52.67% for P1-P3, respectively. These results align with the ThT assay results, indicating a significant reduction of β-sheet-positive high-molecular-weight Aβ42 assemblies by FUS.

Through TEM imaging (Figure 2D, Figure S1), we visualized how FUS affects the fibrillar structure of Aβ42 aggregates. Non-treated 2d control formed dense bundles of fibrils, with intact linkage of thin, needle-like structures at the margins. After FUS exposure, it was revealed that peripheral fibrils fragmented into smaller particles and dispersed, leaving a dense central core.

### FUS-induced disaggregation of Aβ42 oligomers *in vitro*

Because oligomeric Aβ species are considered the most neurotoxic forms of Aβ aggregates, we next assessed whether FUS induced an unexpected increase in oligomer levels due to the fibril disruption. In SDS-PAGE with PICUP chemistry, we revealed that the Aβ42 aggregates with 50~150 kDa (≈10-30-mer; oAβ) decreased by 33.79, 29.16, and 35.36% at P1, P2, and P3, respectively. For another complementary evaluation, dot blot analysis was performed using an oligomer-specific A11 antibody, which is reported to recognize 20~100 kDa oligomers (≈4-22-mer) (Figure 2E, Figure S2) 30. Consistent with SDS-PAGE analysis, A11-positive oligomer levels were shown to be reduced by 39.53, 47.78, and 65.02% after P1, P2, and P3 treatment, respectively. Together, these findings indicate that FUS did not generate additional neurotoxic oligomers by fibril dissociation; rather, it reduced both fibrils and oligomers.

Finally, we evaluated the consequences of the FUS exposure on Aβ cytotoxicity using the SH-SY5Y human neuroblastoma cells (Figure 2F). The SH-SY5Y cells were treated with non-incubated Aβ42, 2-day-incubated Aβ42 aggregates, and FUS-treated Aβ42 aggregates for 12 h. We found that the non-incubated Aβ42 sample, which is capable of rapidly forming toxic oligomers during the subsequent 12-h incubation, reduced the viability to 76.90%. The 2-day incubated Aβ42 with abundant aggregates reduced viability to 81.56%. In contrast, the FUS-treated Aβ42 sample improved the viability to 90.48%, indicating that FUS attenuated the Aβ42-induced cytotoxicity, as well as the Aβ particles generated after FUS exposure do not re-engage into toxic oligomers over the 12-h incubation. Collectively, we revealed that FUS treatment disrupts Aβ42 aggregates *in vitro*, especially β-sheet-rich fibrils and oligomers, thereby reducing their cytotoxicity.

### Dissociation of amyloid plaque by FUS treatment *ex vivo*

For the experiment, 14-month-old 5XFAD mice were sacrificed, and brain tissue was sectioned into 25-μm-thick slices. To directly compare the effects of FUS, the two brain slices were placed symmetrically, each with the same cut surface facing upward (Figure 3A). The tissue was positioned on a polymer-bottomed dish to minimize reflection of acoustic stimulation, with the hippocampus centered. Then the dish was placed on the transducer guide, topped by an ultrasound absorber (Figure 3B). The system was filled with degassed water, and sonication was applied for 30 min using the parameters detailed in Figure 3B. Sonication parameters were selected based on previous studies that demonstrated an effective streaming effect on brain structure induced by sonication 31-33.

**Figure 1:**
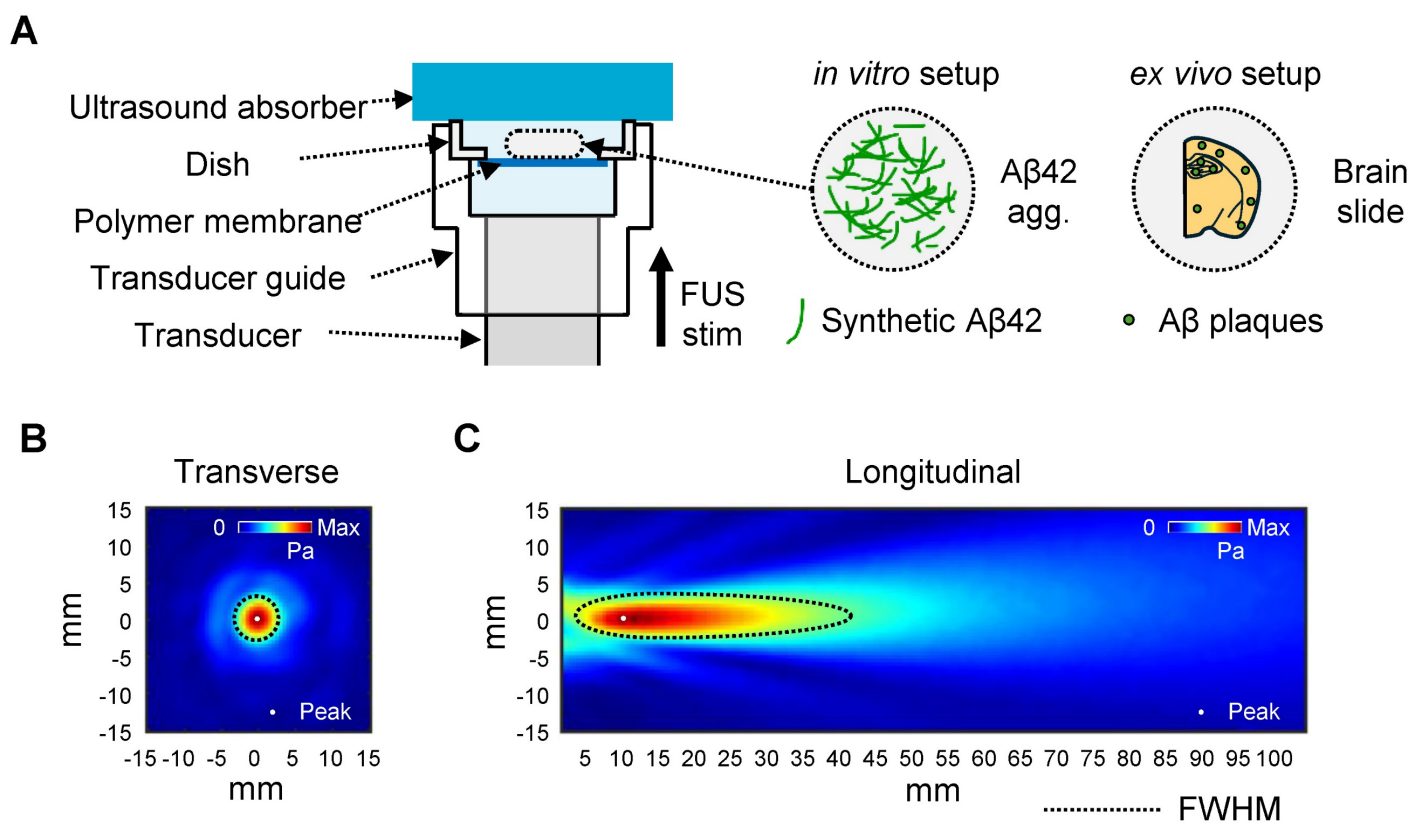
***In vitro* and *ex vivo* experimental setup.** (**A**) Schematic diagram of the FUS-induced synthetic Aβ42 or brain slice stimulation setup. (**B-C**) Beam profile of the FUS stimulation along the transverse (**B**) and longitudinal (**C**) planes of the focus. The dotted line indicates the FWHM, and the white dot indicates the location of the peak pressure. stim, stimulation; agg., aggregates.

**Figure 2:**
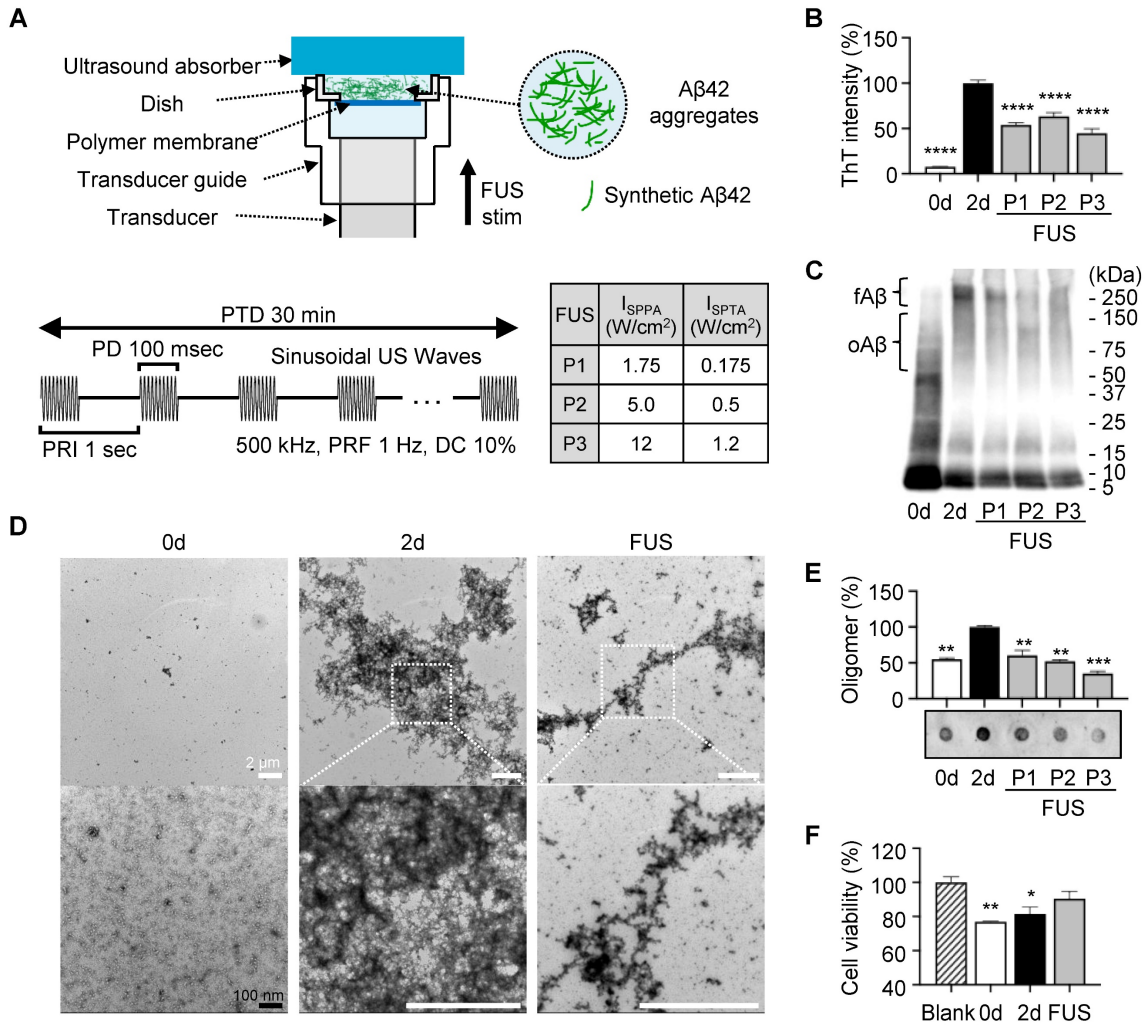
** Dissociation of Aβ42 fibrils and oligomers by FUS *in vitro*.** (**A**) Schematic diagram of the FUS-induced synthetic Aβ42 aggregates stimulation setup and temporal features of FUS sonication parameters. (**B**) ThT fluorescence intensities of FUS (P1-P3)-treated Aβ42 samples with non-incubated (0d) and 2-day incubated (2d) Aβ42 controls. (**C**) Western blot analysis of Aβ42 samples using 6E10 (1:10,000) antibodies. Aggregates around 250 kDa (≈55-mer) and 50~150 kDa (≈10-30-mer) are marked as fibrillar Aβ42 (fAβ) and oligomeric Aβ42 (oAβ). (**D**) Representative TEM images of 0d, 2d, and FUS-treated Aβ42 samples. More images are presented in Figure S2. Magnified images of white dashed squares are placed in the row below. White scale bars represent 2 μm, and a black scale bar represents 100 nm. (**E**) Relative oligomer levels detected using A11 antibody (1:1,500). (**F**) Viability of SH-SY5Y cells exposed to 0.2% DMSO in DMEM media (blank), 0d, 2d, and FUS-treated Aβ42 samples was evaluated using the CCK cell viability kit (Eubiogene, Korea). One-way ANOVA followed by Bonferroni's multiple comparisons test was performed for statistical analyses (*p < 0.05, **p < 0.01, ***p < 0.001, and ****p < 0.0001 vs. 2d in **B**, **E**, and Blank in **F**). Data are presented as the mean of triplicated experiments ± SEM. stim, stimulation.

**Figure 3:**
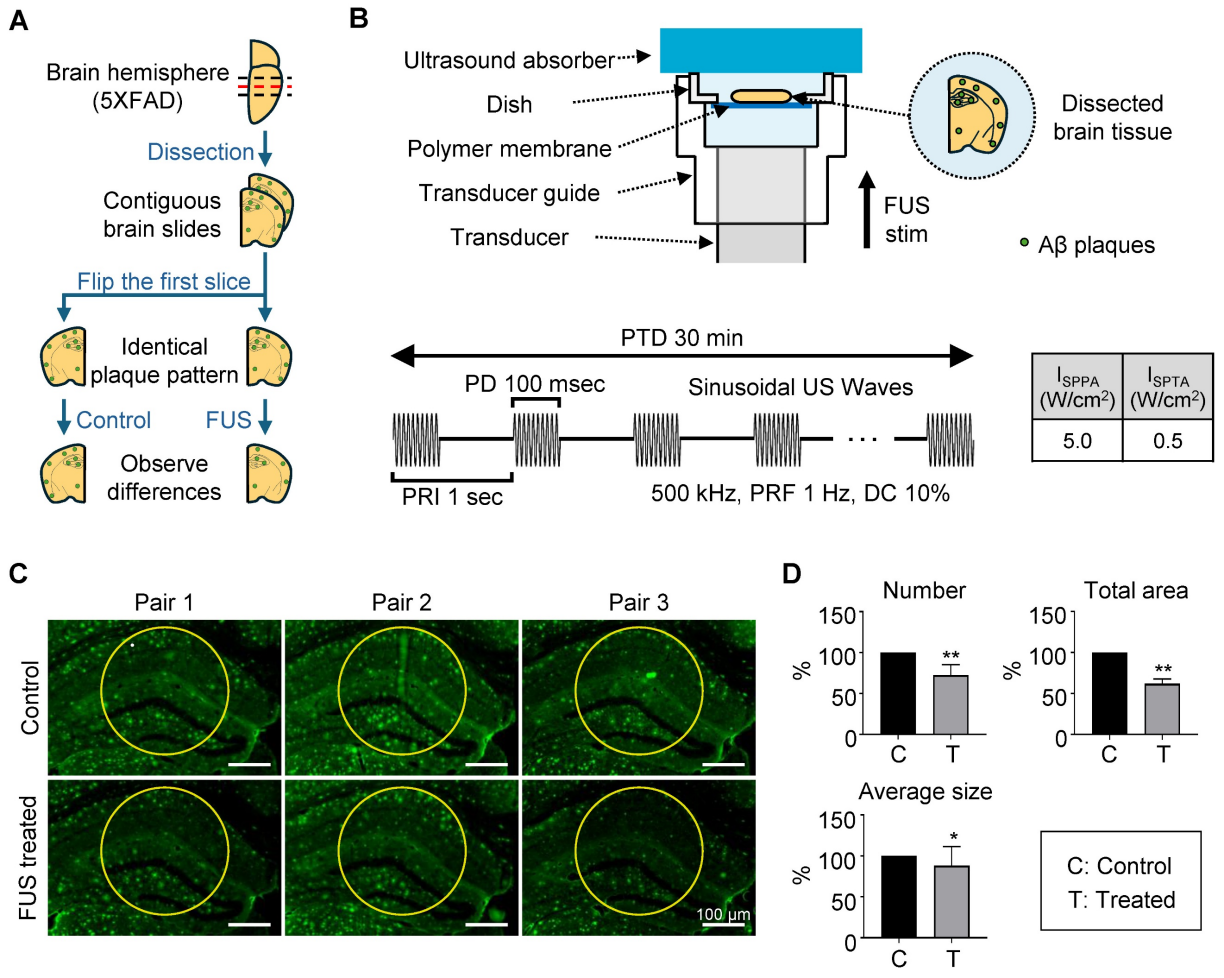
** Dissociation of amyloid plaque *ex vivo*.** (**A**) Brain sample preparation scheme. The red dashed line indicates the adjacent surface of two consecutive slices, which share an identical plaque pattern and were used in the experiment. Green dots on the brain slide indicate amyloid plaques. (**B**) Schematic diagram of the FUS-induced brain slice stimulation setup and temporal features of FUS sonication parameters. (**C**) Brain tissue image after immunohistochemistry using the 6E10 antibody. Yellow circles indicate the analyzed area, with a diameter of 300 μm. (**D**) Comparison of plaque number, total area, and average size between control and FUS-treated brain tissue. Statistical analysis was performed using an unpaired t-test (*p < 0.05 and **p < 0.01). Data are presented as the mean of triplicated experiments ± SEM. stim, stimulation.

The center of the sonicated region with a diameter of 300 µm of the tissue image was analyzed for measuring the Aβ plaques. (Figure 3C, each area enclosed by the yellow circle). This point was indicated in the acoustic beam profile (Figure 1B-C, white dot), which shows the center of acoustic beam stimulated the target region. Our immunohistochemistry results show that a significant decrease in the number, total area, and average size of the plaques in the hippocampal region was observed after FUS sonication (Figure 3D), compared to the control condition.

### Awake tFUS stimulation

**Figure 4:**
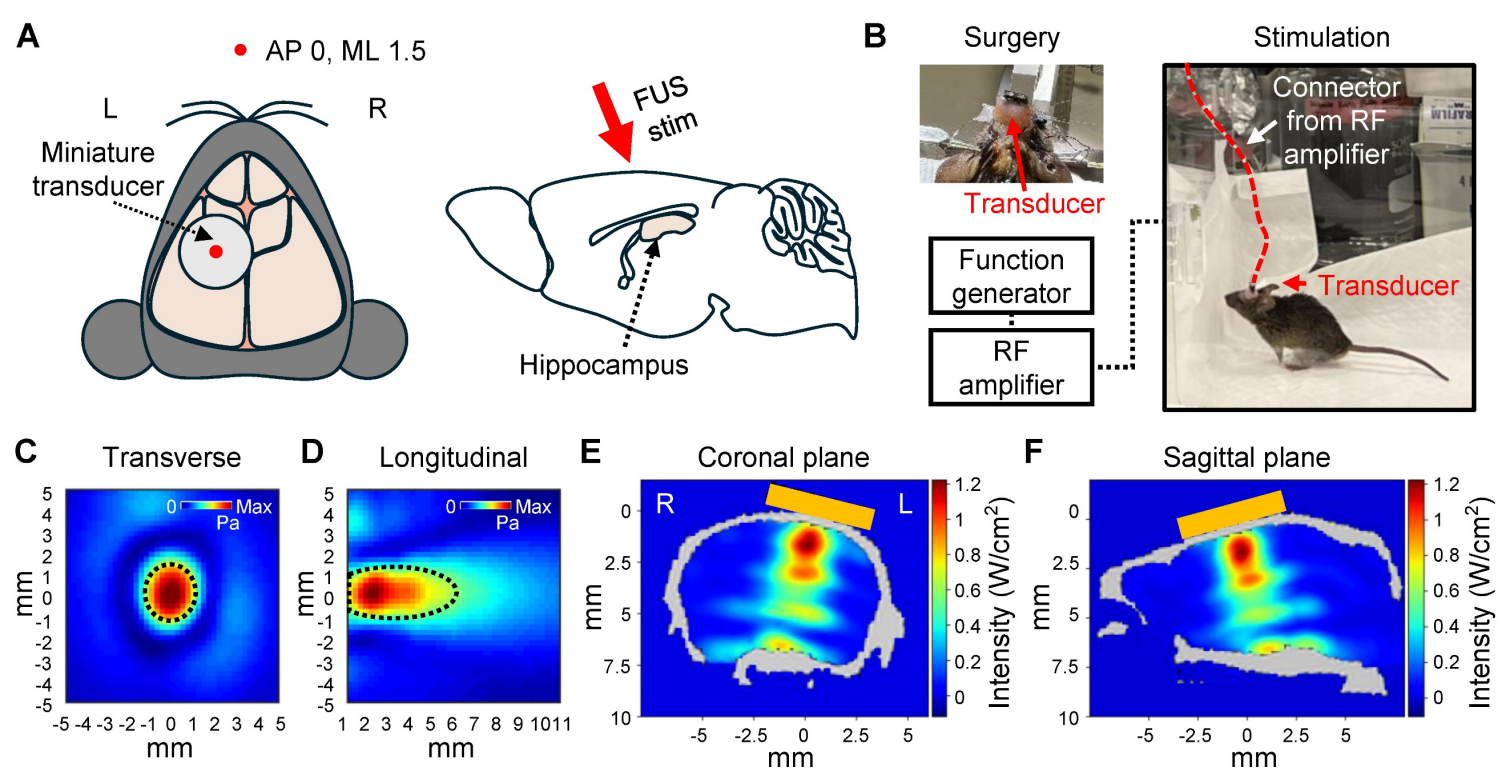
** Setup of *in vivo* awake tFUS stimulation.** (**A**) Schematic diagram of the transducer fixation (left) and sagittal view (right) of the brain. The red arrow indicates the direction of the tFUS stimulation. (**B**) Example image of the transducer fixation surgery and *in vivo* awake stimulation in the mouse. (**C**-**D**) Beam profiles of the FUS stimulation along the transverse (**C**) and longitudinal (**D**) planes of the focus. The dotted line indicates the FWHM. (**E**-**F**) Acoustic simulations of the tFUS stimulation. 2D cross-sectional view from numerical simulation of coronal (**E**) and sagittal (**F**) planes is visualized. Orange box indicates the position of the transducer on the skull. L, left hemisphere; R, right hemisphere.

We built a system for *in vivo* awake tFUS stimulation for 5XFAD mice to investigate the effect of sonication for Aβ plaque dissociation and clearance (Figure 4). A miniaturized transducer specifically designed for *in vivo* tFUS procedures in awake animals was employed. The transducer had a center frequency of 450 kHz and a diameter of 5 mm. Details regarding the fabrication of the transducer are available in our previous work 34.

The transducer was affixed to the skull to target the left hippocampus (Figure 4A), allowing comparison with the contralateral hippocampal region to assess the effect of sonication. Following the procedure, the mice were given a one-week recovery period to allow the surgical site to heal and to ensure stable attachment of the transducer. As shown in Figure 4B, the animals were freely movable during the 30-minute stimulation session with the transducer and a lightweight connector.

The beam profile of the transducer (Figure 4C-D) and acoustic simulations (Figure 4E-F) demonstrated that sonication was focused on the target region. The parameters for FUS stimulation, such as PRF, PRI, and PTD, were consistent with the *ex vivo* experiments. However, the operating frequency was set to 450 kHz to match the transducer's center frequency, and the acoustic intensity was reduced (1.75 W/cm^2^ I_SPPA_ and 0.175 W/cm^2^ I_SPTA_) compared to the *ex vivo* experiment.

The intensities of FUS stimulation were selected to ensure safe brain stimulation according to the FDA's safety guidelines 35 while adapting them to the experimental conditions of the animal model. Considering the acoustic attenuation through the human skull (Fz region of the international 10-10 EEG standard system) 36, a stimulation intensity of 5 W/cm² I_SPPA_ and 0.5 W/cm² I_SPTA_ applied to the human brain is estimated to correspond to approximately 1.25 W/cm² I_SPPA_ and 0.125 W/cm² I_SPTA_ at the target site. This estimated intensity is consistent with the *in situ* intensity derived from our acoustic simulations.

### Dissociation of amyloid plaque by FUS treatment *in vivo*

**Figure 5:**
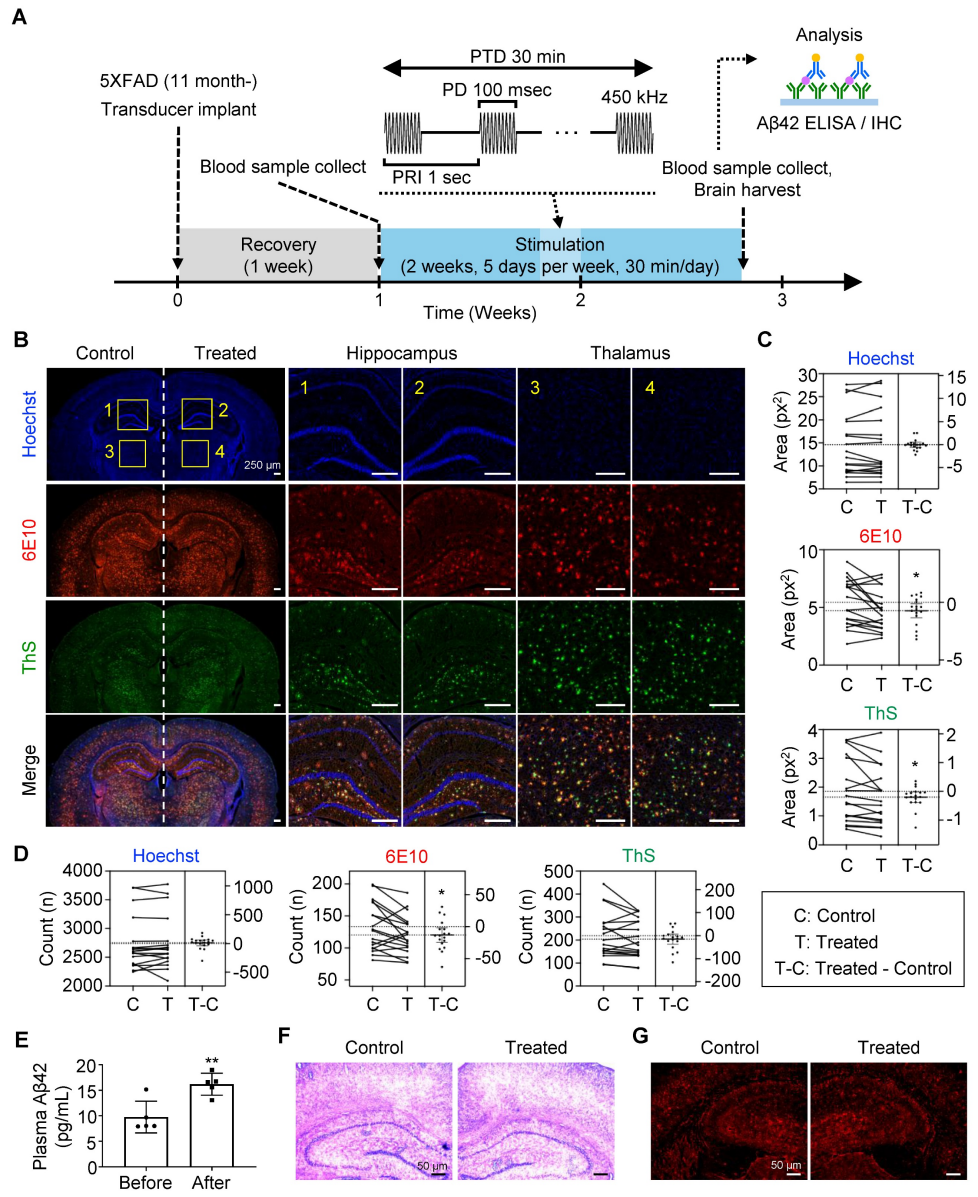
** Dissociation and clearance of amyloid plaque *in vivo*.** (**A**) Experimental timeline and flow of the *in vivo* plaque dissociation experiment. (**B-D**) Immunohistochemistry of brain slides for neuron (Hoechst), total plaque (6E10), and dense core plaque (ThS). (**B**) Representative brain images showing hippocampus (1, 2) and thalamus (3, 4), which were used for neuronal and plaque quantification. Comparison of the plaque (**C**) size and (**D**) number between the FUS-treated (Treated, T) and untreated (Control, C) sides of the brain slice. (**E**) Comparison of plasma Aβ levels before and after FUS treatment. (**F**) Representative brain tissue image after hematoxylin and eosin staining. (**G**) Representative brain tissue image after immunohistochemistry with GFAP antibody. Statistical analysis was performed using a paired t-test in **C**, **D**, and an unpaired t-test in **E** (*p < 0.05 and **p < 0.01). IHC, immunohistochemistry.

It was previously reported that mechanically dissociated Aβ plaques were solubilized and cleared via brain-to-blood efflux 37, 38. To confirm the outflow of Aβ by FUS, we collected plasma from the lateral saphenous vein before FUS treatment (Figure 5A). Each mouse then underwent two weeks of FUS treatment, consisting of five consecutive days of 30-min FUS exposure, a two-day rest period, and another five days of FUS treatment. After the final FUS treatment for 15 min, plasma was collected from the posterior vena cava for comparison, followed by brain tissue collection for further analysis.

When comparing the Aβ deposits in the FUS-treated sides to the non-treated side of the brain (Figure 5B-D, Figure S3), we found a significant reduction in 6E10-positive plaque number and size (*p* = 0.0284 and 0.0218, respectively) in the targeted hippocampus and the adjacent thalamus by FUS. It was also shown that the size of ThS-positive dense core plaques (*p* = 0.0327), but not the number (*p* = 0.1752), is reduced by FUS treatment. Plasma analysis revealed a 65.91% increase in Aβ42 levels after FUS treatment (from 9.758 pg/mL to 16.19 pg/mL, Figure 5E), suggesting that FUS dissociates Aβ plaques, which are subsequently released into the bloodstream. No signs of hemorrhage or inflammation were observed following FUS treatment (Figure 5F-G, Figure S4), as previously reported that the low-intensity FUS is known to have minimal adverse effects 39. Collectively, we confirmed the disruption and clearance of Aβ plaque by FUS stimulation *in vivo*.

## Discussion

In this study, we investigated the potential of FUS as a tool for Aβ plaque dissociation. We found that FUS treatment induced the disruption of fibrillar and oligomeric Aβ42 *in vitro.* In *ex vivo* experiments, FUS exposure to brain slices from 5XFAD transgenic mice led to a reduction in Aβ plaque numbers, area, and size within the targeted region. Similarly, *in vivo* application of FUS over two weeks resulted in a diminished plaque burden. The observed increase in plasma Aβ42 levels further suggests that the dissociated Aβ plaques were cleared into the bloodstream.

Although the precise interactions within amyloid structures remain unclear, previous studies have shown that β-sheet structures of amyloid proteins are characterized by a network of relatively weak interactions, including various types of hydrogen bonding 40. Based on this feature, we estimated that the application of low-intensity ultrasound mechanical energy may exert sufficient force to dissociate these interactions, potentially leading to the reduction of amyloid plaques. To focus on the mechanical effects of FUS on Aβ aggregates, we performed direct FUS exposure to synthetic Aβ42 aggregates *in vitro*. FUS stimulation from low (1.75 W/cm^2^ I_SPPA_, 0.175 W/cm^2^ I_SPTA_) to high (12 W/cm^2^ I_SPPA_, 1.2 W/cm^2^ I_SPTA_) intensities demonstrated significant breakdown of their β-sheet structure. Next, we adopted stimulation protocols established in previous studies 31, 33. Our *ex vivo* experiments demonstrated that mechanical vibration induced by FUS alone was sufficient to reduce the number of accumulated Aβ plaques in the hippocampal region. This indicates a direct physical effect of FUS on aggregated Aβ structures, independent of any neuromodulatory or pharmacological mechanisms.

In the *in vivo* experiment, we applied a lower intensity (1.75 W/cm^2^ I_SPPA_, 0.175 W/cm^2^ I_SPTA_) to match the estimated *in situ* intensity observed through the human skull when delivering 5 W/cm^2^ I_SPPA_ and 0.5 W/cm^2^ I_SPTA_, thereby facilitating clinical translation. Despite the reduced intensity, we still observed a decrease in both the number and size of Aβ plaques. These results suggest that *in vivo* FUS may exert additional effects beyond direct mechanical dissociation of Aβ plaques, potentially influenced by the structural and systemic environment of the brain. Our previous work using the same PRF, DC, PD, and PTD demonstrated that FUS can enhance CSF circulation, particularly through the perivascular space, and FUS accelerates the movement of nanoparticles that mimic brain waste 33. The parameters used were also effective in transporting particles through porous structures resembling brain tissue, driven by acoustic forces 31. These properties suggest that the FUS protocol may enhance CSF clearance mechanisms. Given the CSF-to-blood waste clearance pathway mediated by the perivascular network 41, the observed elevation in plasma Aβ levels after FUS treatment supports this hypothesis, indicating increased washout of amyloid particles from the brain. Taken together, our *in vivo* results imply a dual mechanism: FUS may first dissociate Aβ aggregates mechanically, followed by enhanced CSF circulation.

FUS has been widely applied in conjunction with microbubbles to safely and reversibly open the BBB 42. This combined approach offers promising synergistic benefits through two principal mechanisms. First, temporary BBB opening facilitates targeted delivery of therapeutic agents that promote Aβ breakdown 17. When paired with FUS-mediated mechanical dissociation, such agents may achieve enhanced efficacy against amyloid pathology. Second, increased BBB permeability may activate endogenous clearance pathways, aiding in the removal of amyloid fragments liberated by FUS 14, 43. These findings collectively underscore the potential of FUS-based strategies as a multifaceted therapeutic approach for AD. However, the possible side effects such as brain edema or microhemorrhage should be considered for applying these combined approaches. Weakened BBB structure of AD patients elevates the risks of opening BBB 44, while FUS dose for mechanical dissociation may require a higher intensity than the safe level for a weakened BBB. For this reason, possible risks should be evaluated while combining BBB opening and enhancing CSF circulation and mechanical dissociation of Aβ plaques. Combined techniques can maximize the reduction of Aβ plaques, while limited and personalized approaches are required, considering personal amyloid pathology and severity.

Beyond vascular effects, microglia play a complex and dualistic role in AD: while contributing to neuroprotection through phagocytic clearance of Aβ plaques, they may also trigger neuronal damage through chronic inflammation 45. FUS has been shown to modulate microglial activity, potentially biasing their function toward a more beneficial, phagocytic phenotype 46, 47. Although further investigation is required to fully understand microglial responses under FUS treatment, the enhanced phagocytic activity observed suggests that microglia may contribute to plaque clearance following FUS stimulation, supporting a therapeutic strategy aimed at mitigating disease progression 48.

In the broader therapeutic landscape, the recent FDA approval of anti-Aβ antibody therapies highlights the potential of amyloid-beta clearance in AD 49. However, these treatments are associated with significant risks, including cerebral edema and hemorrhage, particularly among *APOE4* carriers 50. Moreover, the high cost of antibody therapies presents a challenge for widespread adoption and imposes a burden on public healthcare systems. In contrast, low-intensity FUS has demonstrated a favorable safety profile, and our findings further support its efficacy in reducing amyloid burden. These advantages position FUS as a promising non-invasive alternative to antibody-based therapies.

For the further development of the FUS-mediated Aβ plaques control techniques, personalized dose modulation and stimulation position selection methods can be implemented. We modulated the stimulation dose in consideration with attenuation parameters based on average skull thickness and bone density 36, while moving to the *in vivo* transcranial stimulation experiments. However, the acoustic attenuation rate could vary by the patient's skull thickness, skull shape, age and bone density 51, 52. For this reason, personalized sonication protocol in consideration of intensity and stimulation position would be needed for increasing the accuracy of the ultrasound-based treatments. The development of numerical evaluation and neuronavigation techniques based on neuroimaging data can provide guidance for precise control of the FUS stimulation 53, 54. Although our results demonstrate the efficacy of FUS in reducing amyloid plaque burden, it is important to note that plaque clearance does not necessarily translate into cognitive or behavioral improvement in AD 55. Future studies should evaluate the impact of FUS on cognitive and behavioral outcomes to fully assess its therapeutic potential. Additionally, a more detailed analysis of Aβ species *in vivo*, including oligomeric Aβ, may help elucidate the diverse effects of FUS on amyloid pathology and further validate focused ultrasound as a viable treatment strategy. It needed to validate whether FUS parameters could be effectively engineered to target specific Aβ species, which is closely linked to neurotoxicity.

## Conclusions

FUS effectively reduces amyloid plaques in both *ex vivo* and *in vivo* models, highlighting its potential as a non-invasive strategy for Aβ clearance.

## Supplementary Material

Supplementary figures.

## Abbreviations

*Abb*: Abbreviation
AD: Alzheimer's disease
Aβ: Amyloid-β
AP: Anterior-posterior
APS: Ammonium persulfate
BBB: Blood-brain barrier
CSF: Cerebrospinal fluid
DC: Duty cycle
DMEM: Dulbecco's Modified Eagle Medium
DMSO: Dimethyl sulfoxide
DTT: Dithiothreitol
ELISA: Enzyme-linked immunosorbent assay
Fmoc: Fluorenylmethyloxycarbonyl chloride
FUS: Focused ultrasound
FWHM: Full-width at half-maximum
GFAP: Glial fibrillary acidic protein
HRP: horseradish peroxidase
H&E: Hematoxylin and Eosin
ML: Medial-lateral
PAGE: Polyacrylamide gel electrophoresis
PBS: Phosphate-buffered saline
PD: Pulse duration
PICUP: photo-induced cross-linking of unmodified protein
PTD: Pulse train duration
PRF: Pulse-repetition frequency
PRI: Pulse repetition interval
SDS: Sodium dodecyl sulfate
SEM: Standard error of the mean
SPPS: Solid-phase peptide synthesis
I_SPPA_: Spatial-peak pulse-average intensity
I_SPTA_: Spatial-peak temporal-average intensity
TBST: Tris-buffered saline containing 0.1% Tween 20
TEM: Transmission electron microscope
tFUS: Transcranial FUS
ThS: Thioflavin S
ThT: Thioflavin T
RF: Radio frequency
RuBpy: Tris(2,2-bipyridyl)dichlororuthenium(II) hexahydrate
