# Supplementary material for: Clearance of amyloid plaque via focused ultrasonication in a mouse model of Alzheimer's disease
Source: Theranostics. 2026 Jan 1;16(5):2310–23. doi: 10.7150/thno.122123 (PMC12712922; doi:10.7150/thno.122123)
Supplement: Supplementary file 1 — Supplementary figures. [file thnov16p2310s1.pdf]

Supplementary Figures

Clearance of amyloid plaque via focused ultrasonication in a mouse model of Alzheimer's disease

Songmin Lee[1*], Jeungeun Kum[2*], Kyeonghwan Kim[1*], Tae Young Park[2], HeeEun Ko[2], Duk L. Na[3,4], Suk Yun Kang[5], Hyungmin Kim[2**], YoungSoo Kim[1**], and Jaeho Kim[5**]

[1]Department of Pharmacy and Yonsei Institute of Pharmaceutical Science, College of Pharmacy, Yonsei University, Incheon, Republic of Korea

[2]Bionics Research Center, Biomedical Research Division, Korea Institute of Science and Technology, Seoul, Republic of Korea

[3]Happy mind Clinic, Seoul, Republic of Korea

[4]Department of Neurology, Samsung Medical Center, Sungkyunkwan University School of Medicine, Seoul, Republic of Korea

[5]Department of Neurology, Dongtan Sacred Heart Hospital, Hallym University College of Medicine, Hwaseong-si, Gyeonggi-do, Republic of Korea

*These authors contributed equally to this work

**Corresponding authors

Corresponding author 1: Jaeho Kim

Phone: +82-31-8086-2310 Fax: +82-50-4068-1402 E-mail: ion8484@gmail.com

Co-corresponding author 2: YoungSoo Kim

Phone: +82-32-749-4523 Fax: +82-32-749-4105 E-mail: y.kim@yonsei.ac.kr

Co-corresponding author 3: Hyungmin Kim

Phone: +82-2-958-5695 Fax: +82-2-958-5629 E-mail: hk@kist.re.kr

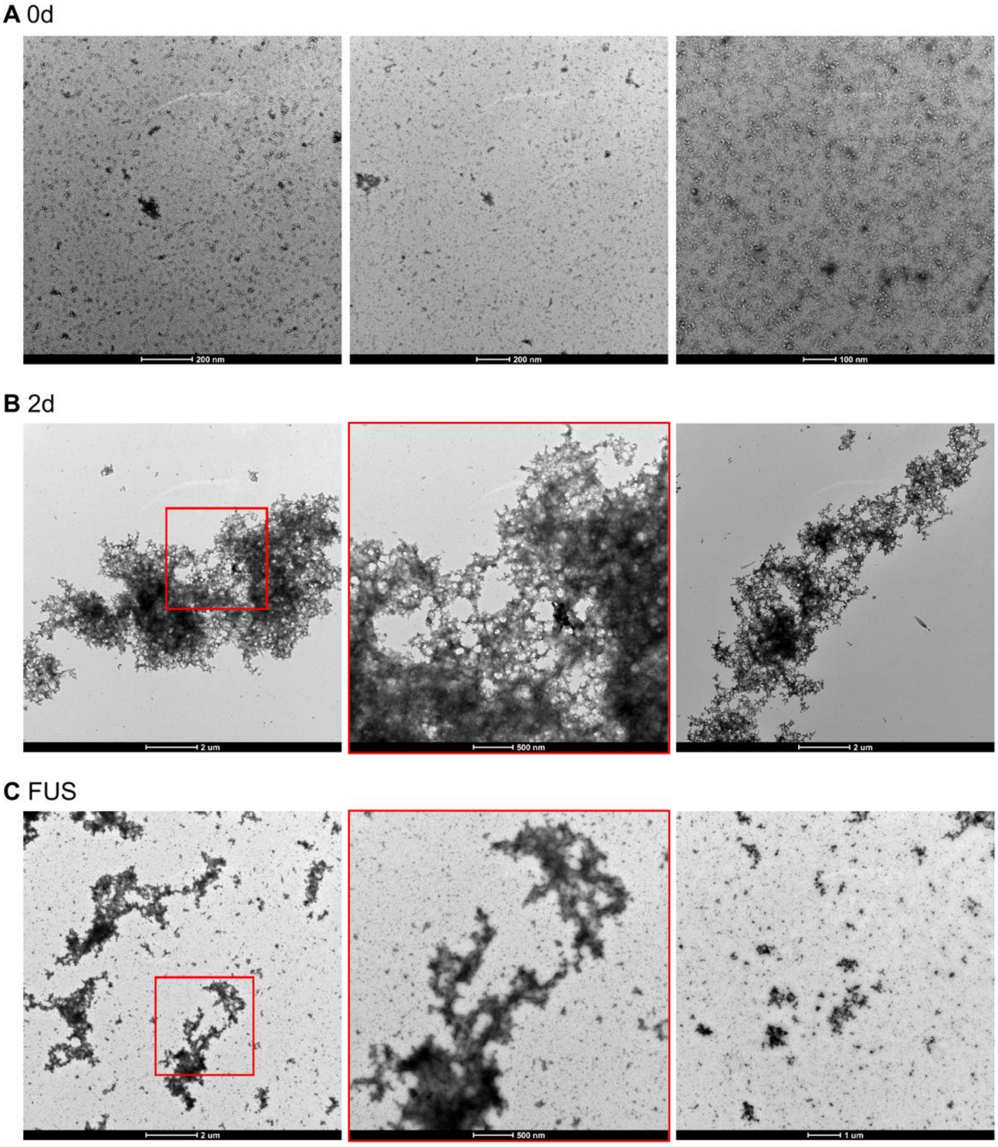

Supplementary Figure 1. TEM images showing FUS-induced disaggregation of Aβ42 fibrils *in vitro*. Representative images of Aβ42 peptides before aggregation (0d), after 2 days of aggregation (2d), and after FUS exposure applied to 2-day aggregates (FUS). A magnified image corresponding to the red square is presented on the right.

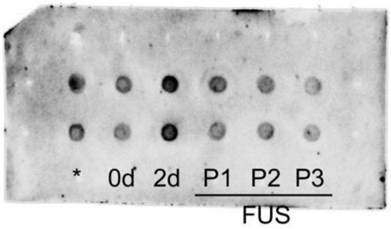

Supplementary Figure 2. Dot blot analysis of Aβ42 oligomers after FUS treatment *in vitro*. The entire membrane image for the dot blot using the A11 antibody (1:1,500). Aβ42 peptides before aggregation (0d), after 2 days of aggregation (2d), and after FUS exposure applied to 2-day aggregates (FUS) were blotted on the nitrocellulose membrane. Asterisk indicates a sample not related to this experiment.

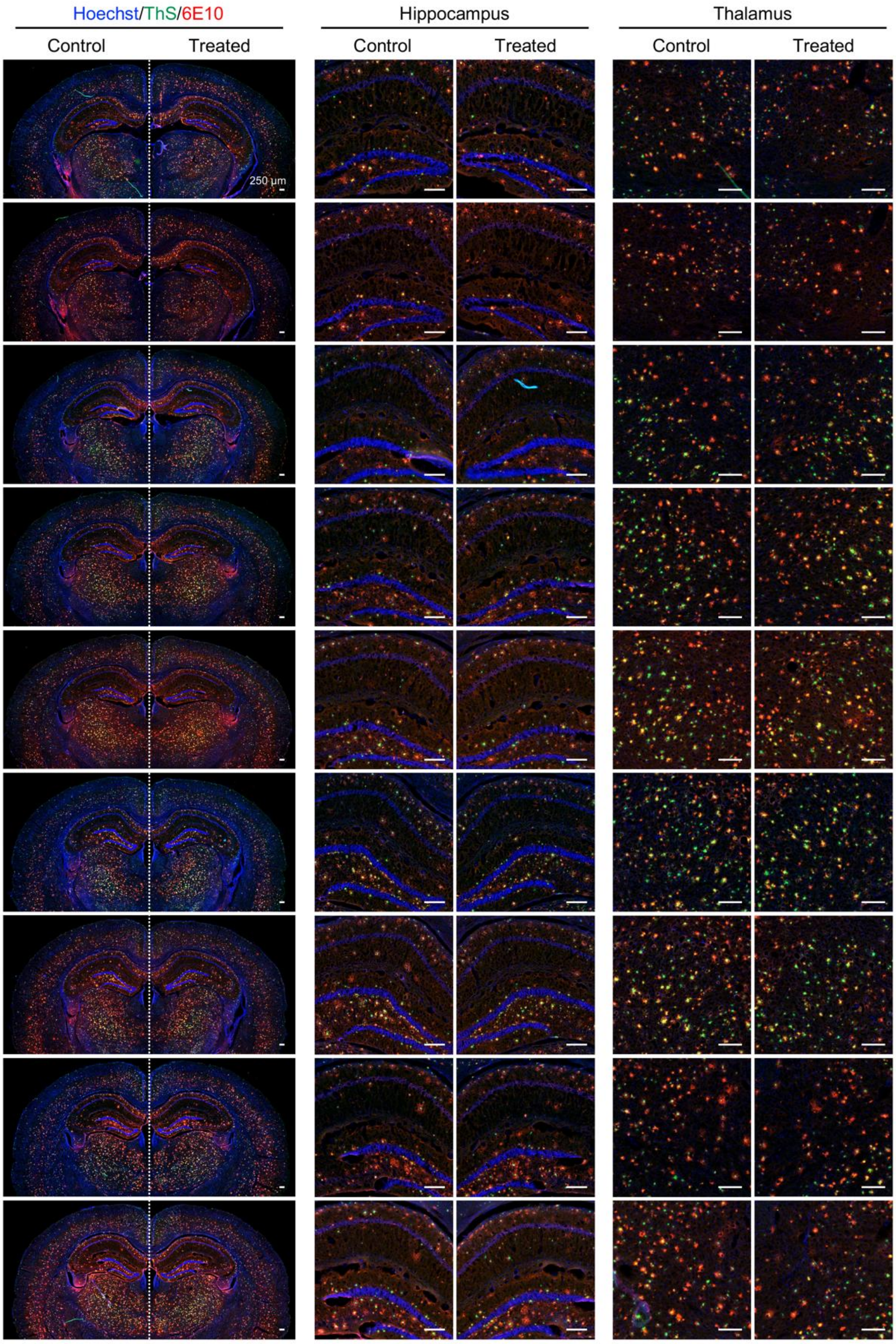

Supplementary Figure 3. ThS and 6E10 co-staining in FUS-treated 5XFAD mice. Two brain sections per mouse were used for plaque counting; however, one section was excluded due to image quality issues. The hippocampal and thalamic regions of non-treated (Control) and FUS-treated (Treated) hemispheres are presented.

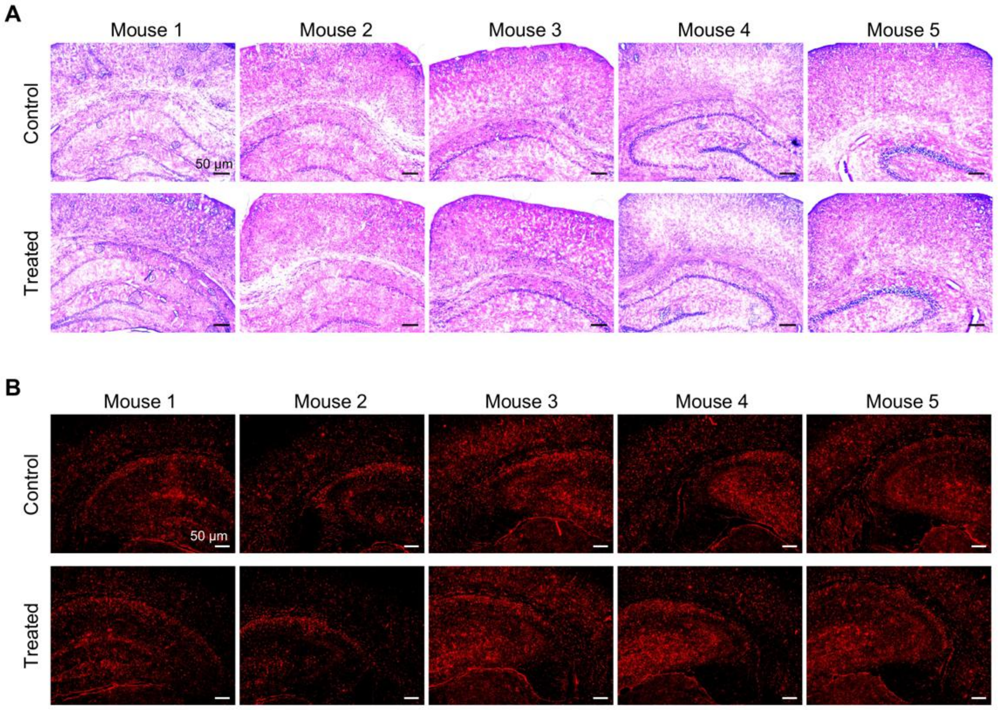

Supplementary Figure 4. H&E and GFAP staining in FUS-treated 5XFAD mice. (**A**) H&E and (**B**) GFAP immunostaining in FUS-treated 5XFAD mice. Whole hippocampal images of the non-treated right hemisphere (Control) and FUS-treated left hemisphere (Treated).
